# Thrombocytopenia with absent radii (TAR) syndrome in a female neonate: a case report

**DOI:** 10.11604/pamj.2019.33.181.13928

**Published:** 2019-07-09

**Authors:** Olayemi Atinuke Alagbe, Adekunle Emmanuel Alagbe, Emmanuel Olugbenga Onifade, Temitope Olugbenga Bello

**Affiliations:** 1 Department of Radiology, Lautech Teaching Hospital Osogbo, Osun State, Nigeria; 2 Department of Haematology, University College Hospital Ibadan, Oyo State, Nigeria

**Keywords:** Thrombocytopenia, congenital, haemorrhage

## Abstract

Thrombocytopenia absent radius (TAR) syndrome is a rare congenital disorder that is consistently associated with skeletal abnormality and thrombocytopenic haemorrhage. This is a case of a neonate with bilateral absent radius and thrombocytopenia. The rarity of this case prompted this report.

## Introduction

Thrombocytopenia with absent radius (TAR) syndrome is a rare autosomal recessive congenital disorder characterised by bilateral absence of radius bone and a reduced platelet count [1]. It has an incidence of 0.42/100,000 live birth and was first discovered in 1951 [2]. TAR syndrome has no gender, ethnic or racial predilection. TARS could be associated with other gastrointestinal, skeletal, hematologic, and cardiac anomalies [3]. Haemorrhage, a major cause of death, occurs commonly in the first 14 months of life. This is a rare case of TAR syndrome with typical presentation.

## Patient and observation

**Figure 1:**
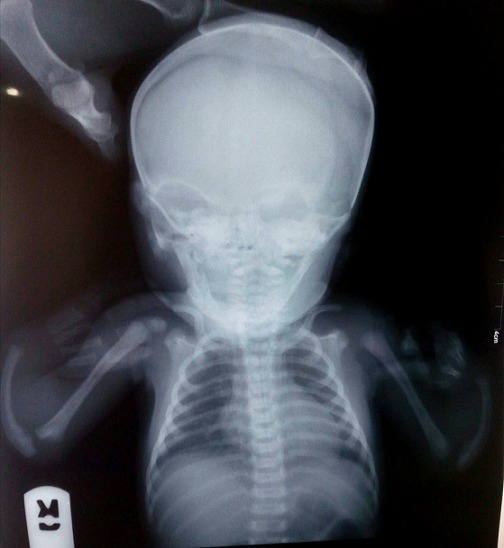
Radiograph of both upperlimbs showing absence of radius bilaterally and ulnar deviation of the hand

A 6-hour old female neonate referred to the radiology department for X-ray of both upper limbs on account of bilateral upper limb deformity from birth. No history of birth trauma, birth asphyxia, family history of similar or other congenital limb deformity. There was attempted termination of pregnancy with the use of a hormonal contraceptive pills (levonorgestrel, dose unknown) and herbal concoctions in the first trimester of pregnancy. She was delivered via spontaneous vaginal delivery at term by a 25-year-old unmarried primigravida. Patient had a birth weight of 2.6 kg, occipitofrontal head circumference (OFC) was 33cm, small anterior frontanelle-1 cm and closed posterior fontanelle. Musculoskeletal system revealed increased tone in the upper limbs compared to lower limbs. No other abnormality was detected in other systems. Plain radiograph of both upper limbs reveals bilateral absent radii, shortening and bowing of the ulna, abnormal flexion and radial deviation of both hands (Figure 1). Other bones of the upper limbs are present and demonstrate no abnormality. No deformity is seen in other parts of the body. Abdominal ultrasound revealed normal abdominal organs. Complete blood count on the first day of life revealed haematocrit 47%, leucocytes of 9600/mm^3^with neutrophils 74%, lymphocytes 26% and thrombocytopenia of 70,000/mm^3^. A repeat blood count on the 14^th^ day of life showed a declining haematocrit of 35% and thrombocytopenia of 65000/mm^3^ and leucocytes remained normal 7000/mm^3^.

## Discussion

TAR syndrome is a rare autosomal recessive disorder. It is an inherited syndromic thrombocytopenia that is consistently associated with skeletal abnormality and thrombocytopenic haemorrhage. It has no gender preference but some studies have reported female's predilection [1]. Like in the index case, 2 previous reports of TAR syndrome by Musa *et al.* and Alao *et al.* from different regions in Nigeria were in females [4,5]. The cause of the thrombocytopenia is still unclear. However, several theories have been proposed. The widely acceptable theory is the failure in production of humoral or cellular stimulators of megakaryocytopoiesis. Alterations of the RBM8A gene involving a microdeletion on chromosome 1q21.1. has been associated as the molecular basis of this syndrome [6,7]. This was however, not assessed in the index case for paucity of facility for molecular studies. Conspicuous in this report is the use of levonogestrel and herbal concoctions content of which was not known. Though ‘morning after’ pills are reported to be safe for both mother and fetus even if the pregnancy is not aborted, an association cannot be ignored, more so, concomitant use of herbal concoction is also implicated. This case report buttresses the teratogenic effect of drugs and chemicals especially during the first trimester as in this case.

The platelet count is frequently less than 50,000 with normal platelet morphology peripheral blood film examination. Other associated haematological features are eosinophilia, leukocytosis with a left shift. The WBC count in this case was normal. Thrombocytopenic episodes are most frequently during the first 2 years of life; this explains why incidence of hemorrhage is common in the first 14 months of life and is the major cause of mortality [8]. Even though hemorrhagic episodes are the most common cause of mortality as reported by Hedberg *et al.* this patient had no thrombocytopenic bleed [8]. This may be due to mild thrombocytopenia in this patient. Spontaneous bleeding is rare with mild thrombocytopenia except with surgery, trauma or associated thrombocythopathy. However, serial monitoring of the blood counts has been recommended for prompt intervention. About 50% of affected infants are symptomatic in the first week of life and 90% are symptomatic by the age of 4 months. Thrombocytopenia can fluctuate over time [7]. Therefore, with strong suspicion TAR syndrome despite initially normal platelet count, serial platelet count monitoring has been recommended. The platelet count in this case showed reduction in platelet count from 70000 to 65000 at 2 weeks interval. With advancing age, the recurrence of thrombocytopenic episodes reduces. There is need for counselling of parents to avoid trauma and intramuscular injections so as to prevent haemorrhage in the patient. With continuous reduction of platelet count, patient may benefit from transfusion with platelet concentrate, packed cells and or stem cell transplantation later in life. As part of management, physiotherapy would be required. Mental retardation is associated with about 7% of all cases of TAR syndrome. This is probably sequel to complications from intracranial hemorrhage caused by thrombocytopenia [9]. Structural defects that predispose the patient to mental retardation and other neuropsychiatric disorders (psychosis) have been proposed [10]. This index patient has closed posterior fontanelle and small anterior fontanelle. Only patients with TAR syndrome consistently have bilateral absence of the radii with the presence of thumbs and 4 digits. Upper-extremity function is usually good if radial aplasia is the only skeletal abnormality of the upper extremities.

## Conclusion

Bilateral absence of radii and thrombocytopenia with or without other congenital malformations are the major criteria for the diagnosis of TAR syndrome. The thrombocytopenia fluctuates and the presence of haematological complications depend on the severity of the thrombocytopenia. The aetiology, though unknown, is traceable to drugs and medications taken by the mother during first trimester. Serial monitoring of the platelet count is essential in the management of patients, even in cases with initially normal platelet count.

## Competing interests

The authors declare no competing interests.
